# The practice and prospect of smart cities in China’s urbanization process

**DOI:** 10.1007/s44243-023-00007-w

**Published:** 2023-03-14

**Authors:** Shifu Wang, Dantong Chen, Lianbi Liu

**Affiliations:** School of Architecture, South China University of Technology, Guangzhou, China

**Keywords:** Smart city, China’s urbanization, Smart society

## Abstract

With the rapid development of information technology, the construction of smart city in China has become an indispensable part and support force of new urbanization. By reviewing the practice of smart city in China and reflecting on the experience of smart city construction, this paper puts forward that the construction of smart city should go beyond the perspective of technological progress to build a people-oriented society, and proposes the forward-looking construction of smart city in the new era: building a more intelligent urban society in the process of China’s urbanization.

## Introduction

China’s economy has dramatically changed over the last 40 years of reform and opening-up. After the first breakthrough 50% in 2011, the urbanization rate reached 60% in 2019, still in the rapid development stage. This unprecedented urbanization process has brought opportunities and challenges to city development. On the one hand, traditional urbanization emphasizes on land and population growth while ignoring the balanced and coordinated sustainable development between economic efficiency and urban expansion (Li & Shang, 2014). On the other hand, informatization is having a negative effect on modern urban production and lifestyle, posing new demands on governance optimization. Several “Smarter” strategies are required in administration, social governance, economic development, and cultural revitalization to respond to issues and challenges of China’s urbanization and achieve healthy and sustainable development, signifying the arrival of the era of “Smart Cities.”

Since the submitting of the new-type urbanization in the 18th National Congress of the Communist Party of China, the simultaneous development of industrialization, informatization, urbanization, and agricultural modernization has been the strategic task of present China. The Smart City, which is the most cutting-edge exploration of urban development modes, has become an essential and indispensable part and force of the new-type urbanization with the rapid development of modern Information Technology (Wang & Du, 2014). The development of the Smart City will help in promoting the effective utilization of resources and innovative application in urban development, which will have a great strategic effect on city economic transformation, improvement of the people’s living standards, environmental protection, and management (Min & Chen, 2018). The International Business Machines Corporation (IBM) proposed the Smart City Breakthrough in China” for the first time in 2008 (Qin et al., 2010). Later on, China announced the first batch of national Smart City pilots in 2012 and began to explore the development experience suitable for China’s national conditions. Smart City has evolved from an “exotic” concept to a critical tool for promoting China’s new-type urbanization in the last 10 years. Statistics show that Smart City development in China has advanced and achieved specific goals, although it is still in its early stage, with some deficits in the application and governance processes (Ge, 2017).

As the Chinese socialists entered a new period, technical breakthroughs, mostly typified by informatization, ushered in a new phase of urbanization in China. Based on 40 years of reform and opening up, economic and social development must keep up with the changing world and respond to the new features, such as the transformation of social contradictions, the new normal of economy and society, the middle and late stages of industrialization, the transformation of urbanization, the advent of the information society, and population aging (Li, 2017). These phenomena signify that the development of our cities and society has entered a critical period of transformation. By the end of 2019, China’s urbanization rate of permanent residents has exceeded 60% for the first time (Li, 2020), indicating that urban society had become the fundamental characteristic of the new era. This tremendous change has immensely contributed to the comprehensive development of China’s economy and society. China’s Smart City development has progressed to a new stage with the arrival of the new era. The connotation of the Smart City should not only be limited to the application of knowledge and technology at the level of urban construction but also include a comprehensive penetration in regional development and urban society and point to the future vision and regional relations. Therefore, it is of great significance to reflect on and summarize the practical experience of Smart City development and to provide indications to future development.

## The origin and features of smart city

The concept of Smart City originated from the “Smarter Planet” vision. In November 2008, IBM, the world’s largest global Information Technology consulting firm, presented the ideas of Smarter Planet” at the Council on Foreign Relations in New York, proposing to fully use Internet Information Technology in different industries and realize refined and dynamic management of people’s production and lives to achieve global wisdom (Li et al., 2011). This proposal stimulated widespread social repercussions. Different cities conducted relevant studies and applications. In the same year, IBM proposed the Smart City Breakthrough in China” strategy and signed the co-development agreements with more than 10 provinces and cities in China. New concepts, such as “Smart Planet” and “Smart City”, have attracted widespread attention for a while and have become a new urban development trend. The term “Smart City” has a variety of definitions in the literature, but it can be divided into two levels. On the one hand, many scholars interpret the concept of “Smart City” from a technical perspective, as presented by IBM.

“Smart City” is defined as making full use of Information and Communication Technology to detect, analyze, and integrate the key information used in the core system to support urban operation and to make an intelligent response to various needs, including people’s livelihood, environmental protection, public safety, urban services, industrial and commercial activities, and create better urban life for human beings”, according to IBM’s White Book” from 2008 (Center on Governance, 2003). Based on IBM’s concept, Li suggested that Smart City = Digital City + IoT (Internet of Things) + Cloud Computing”, which involves establishing a smart management and operation mode that is visible and measurable based on the comprehensive digitalization of the city (Li, 2011). Wang et al. believed that Smart City is a smarter city supported by IoT and Cloud Computing that could provide the intelligent response and decision-making to support the operation of the city in every aspect, the essence of which is to make people, as the subject of cities, smarter (Wang et al., 2011). Based on the theory and method of intelligence science, Gong pointed out that a Smart City is the optimal allocation of urban resources through detecting and processing of urban data to ensure high quality and efficient social operation and realize sustainable development (Gong, 2015). European, American, and certain East Asian nations have already implemented the Smart City development plan and gathered important expertise. Many European communities have framed Smart City development as a defined action plan that fits the city’s growth goal using modern information and communication technology. However, the practice has flaws, such as a high imbalance in the interaction between different subjects, and the citizen was relegated to a secondary position (Wu & Bo, 2014; Wu & Lu, 2015). The future development of the Smart City will inevitably focus more on citizens’ demands to their intelligence, with “intelligence” being used to stimulate the potential developments of the economy, society, culture, and ecology.

On the other hand, “Smart City” refers to sustainable urban development. As described by Xu, the genuine Smart City not only enhances the economic and political strength of a city but also promotes social and cultural prosperity and achieves the sustainable development of the economy, society, and environment (Xu et al., 2012). Qiu further proposed that the ultimate purpose of Smart City development is to make a better life for citizens to achieve “good governance” by using modern techniques (Qiu, 2013). Thus, the intellectualization that focuses only on Information Technology application is only one aspect of Smart City, or an approach of realizing Smart City, and it cannot fully represent the connotation of the Smart City. The future development of Smart Cities should be people-oriented, and more attention needs to be paid to the development issue that the city is facing, as well as to the purpose of use innovative technology to achieve sustainable development and build a place for people.

Therefore, we can summarize the definition of Smart City as an emerging future city development approach that is supported by Information and Communication Technology, adheres to the principle of people-oriented and quality-oriented, and aims to achieve sustainable development and a better life.

Based on relevant researches (Wang, 2021), four basic features of Smart City can be summarized as follows: information perception, application integration, interconnection, and sustainable innovation.

1. Information perception: The establishment of the IoTs and the Internet and the use of Information and Communication Technologies allow the comprehensive and effective sensing, collection, processing, and feedback of urban information to ensure the healthy and efficient operation of cities.
2. Application integration: A highly integrated Information Infrastructure is formed to realize unified control and deployment of information and ensure the harmonious and orderly development of the city through the integration, fusion, and assimilation of multiple system information data and applications.
3. Interconnection: Based on the Information Infrastructure, the information and data of various systems in the city can be connected, interacted, and shared by multiple parties to achieve interconnection and intercommunication, promote the efficient collaboration of various key systems and participants in the city, and achieve the best state of city operation.
4. Continuous innovation: Individuals, organizations, enterprises, and governments can rely on the interconnection and intercommunication between Information Infrastructure and the key systems to extensively develop innovative applications of technology and business to provide a steady stream of power for urban development.

## Practices of smart cities in China

### Smart city promotes the development of China’s newtype urbanization

Since 2012, China has been in a phase of economic development that is driven by high-speed urbanization and is supported by the application of emerging Information Technologies. The urbanization rate of the resident population has exceeded 60% for the first time by the end of 2019. The goal of new-type urbanization is to break the urban–rural dualistic conflict and promote modernization. While the Internet has enabled the world to achieve flat connectivity and information equality by reducing information asymmetry barriers (Zhang, 2013), it can be said that urbanization and informatization influence and promote each other.

The Smart City facilitates the deep integration of informatization, industrialization, and urbanization as a vital tool for cities to cope with urban development challenges and achieve innovative transformation (Min & Chen, 2018). The Ministry of Housing and Urban–Rural Development, PRC released “Notice on Pilot Work of National Smart City” in 2012 stating that the development of the Smart City is an important measure to achieve innovation-driven development, promoting new-type urbanization and building a moderately prosperous society, as advocated by the CPC Central Committee and the State Council. The first batch of the National Smart City included 90 pilot cities, followed by the second batch of 103 cities announced in 2013 and the third batch of 97 in 2015. In 2012, the National Smart City pilot mainly concentrated on three coastal regions, namely, the circum-Bohai region, Yangtze River Delta, and Pearl River Delta. Pilot cities began to move inland from coastal regions in 2013 and covered most of the cities in southeast China by 2015. This distribution of cities is similar to the “HU Huanyong Line” distribution law, with a regional imbalance. In 2017, the “Analysis Report on Evaluation Data of National New Smart City” issued by the Secretariat of the Inter-Ministerial Coordination Working Group Office of New Smart City Development and the China Smart City Development Research Center of the State Information Center pointed out imbalances in the regional development and indicated that the collaborative development of city clusters can be considered in the future development of the Smart City to avoid creating city-level silos.

China’s Smart City development has entered a rapid development stage when the Ministry of Housing and Construction announced the National Smart City pilot list, other ministries have also responded, and different types of pilots have been set up. The total number of Smart City-related pilot projects has reached 749 in 2019 (Foresight Industry Research Institute, 2020) (Table 1). Although this development trend is significant in number, multiple in type, and wide in distribution, the development of the Smart City in China has been mainly focused on the technical level due to the initial propaganda influence of IT vendors, such as IBM. However, the Smart City in the developed countries is more a vision of the ideal future city-state for their current situation than a city development model based on specific technologies (Wang & Du, 2014).

**Table 1:** Overview of pilot projects related to “Smart Cities” in China over the years

Year	Name of Ministry	Name of Pilot Projects	Type of Pilot Projects	Number of Pilot Cities	Cumulative Number of pilot Cities
2012	Ministry of Housing and Urban–Rural Development	Pilot Project of National Smart City (First Batch)	Smart Cities	90	90
2013	Ministry of Housing and Urban–Rural Development	Pilot Project of National Smart City (Second Batch)	Smart Cities	103	368
	Ministry of Science and Technology & National Standardization Management Committee	Technology and Standard of Smart City Pilot Project	Technical standard	20	
	Ministry of Industry and Information Technology	National Information Consumption Pilot Project (First Batch)	Specialized	68	
	Ministry of Industry and Information Technology	Pilot project of demonstration of public platform for e-government based on cloud computing	Specialized	77	
	The State Bureau of Surveying and Mapping	Smart City Space-Time Information Cloud Platform Pilot Project (First Batch)	Platform	10	
2014	National Development and Reform Commission	National Pilot City of People-Benefiting Information Construction	Specialized	80	487
	Ministry of Industry and Information Technology & National Development and Reform Commission	2014 Broadband China Demonstration Cities (Urban agglomerations)	Specialized	39	
2015	Ministry of Housing and Urban-Rural Development	Pilot Project of National Smart City (Third Batch)	Smart Cities	97 (special pilot is not included)	694
	Ministry of Industry and Information Technology	National Information Consumption Pilot Project (Second Batch)	Specialized	36	
	Ministry of Industry and Information Technology	National Information Consumption Pilot Cities	Specialized	25	
	The State Bureau of Surveying and Mapping	Smart City Space-Time Information Cloud Platform Pilot Project (Second Batch)	Platform	10	
	Ministry of Industry and Information Technology& National Development and Reform Commission	2015 Broadband China Demonstration Cities (Urban agglomerations)	Specialized	39	
2016	Ministry of Industry and Information Technology & National Development and Reform Commission	2016 Broadband China Demonstration Cities (Urban agglomerations)	Specialized	39	733
2017	The State Bureau of Surveying and Mapping	A New Round of Smart City Space–Time Information Cloud Platform Pilot Project	Platform	46 (includes the pilots of first two rounds)	759
2019	Ministry of Industry and Information Technology	National Information Consumption Pilot Cities	Specialized	15 (adjustments to the 2015 list)	749

Source: provided by the author

**Fig. 1:**
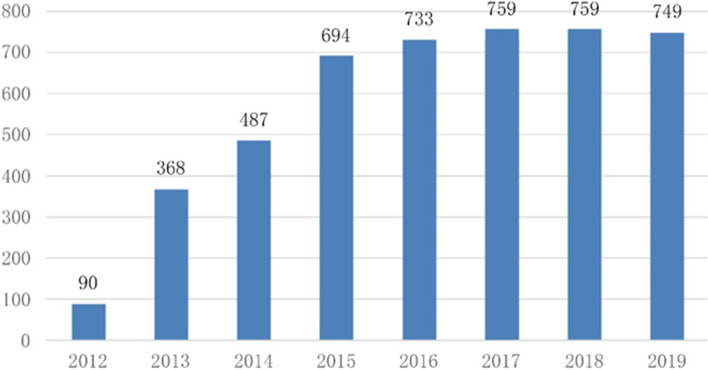
Cumulative number of pilot projects related to Smart Cities” in China over the years. Source: provided by the author

In March 2014, the CPC Central Committee and the State Council issued the “National New-Type Urbanization Planning (2014–2020)”, clarifying that Smart City has become an essential part of new-type urbanization. They pointed out that the development of Smart Cities requires attention on other aspects, such as public service, industrial development, and social governance, in addition to Information Infrastructure. Eight ministries and commissions, including the National Development and Reform Commission, the Ministry of Information Industry, the Ministry of Science and Technology, and the Ministry of Housing and Urban–Rural Development, issued the “Guiding Opinions on Promoting the Healthy Development of Smart Cities” in August of the same year, with the approval of the State Council. According to the guideline, China should build up a batch of Smart Cities that have enhanced radiate effect, improved competitive advantage and distinctive regional characteristics, and achieved satisfactory results in improving people’s livelihood, maintaining information security and innovating social governance by the end of 2020. After 2015, the movement of Smart City pilot works slowed down, and the focus of work shifted from “quantity” to “quality” and from technology application to social governance (Fig. 1). This situation coincides with China’s urbanization process. When the urbanization process has turned from high speed to high quality, the development of Smart Cities has also shifted from promoting economic and technological development to focusing on people and society itself, reverting to the original pursuit of quality and the vision of a better life.

The outbreak of COVID-19 in 2020 has affected and challenged urban operations and development. This pandemic has also brought new ideas to and accelerated the pace of Smart City development. During the pandemic, many cities used new Information Technology to build a smart platform, which played an essential role in social governance, pandemic prevention and control, information dissemination, livelihood security, online learning, and collaborative work. For example, the “Health Code” is a shared service with multiple databases. This platform forces local governments to speed up data integration and break the information isolation. However, problems, such as information privacy and data security, still persist behind the application of Information Technology (Lu, 2020). We need to accelerate the planning and development of Smart Medical Care, Smart Communities, and Smart Logistics to cope with the pandemic. The development of Smart Cities will usher in a period of rapid growth in the future, as part of the development of new infrastructure”, and the normalization of pandemic prevention and control will also bring opportunities for Smart City Technology to expand further and applied.

### Construction and development of typical smart cities

In September 2010, Ningbo was the first to propose Smart City development, followed by Beijing, Shanghai, Guangzhou, Nanjing, and Shenzhen. Several cities entered the substantive stage of Smart City development and achieved great success in improving the living environment, alleviating traffic congestion and improving the efficiency of energy use.

The comparison of the development of typical Smart Cities showed that all cities have introduced relevant policies and plans, most of which are synchronized with the “Five-Year Plan for National Economic and Social Development” to further ensure that the development of Smart City is coordinated with the city’s own development, and have formulated a clear developing strategy. The good economic development conditions and continuously optimized industrial structure of cities have laid the foundation for the development of Smart City. The key measures that cities adopted in the Smart City development during the “13th Five-Year Plan” are analyzed (Table 2). The results also shows that the cities have similar concerns in economic industry, infrastructure construction, traffic management, public services, and social governance. Relevant measures are proposed, although the focus varies according to the economic, social, spatial, and technological conditions of different cities. For example: Beijing and Guangzhou, as leaders of “Beijing–Tianjin–Hebei Urban Agglomerations” and the “Pearl River Delta Urban Agglomeration”, expand their vision from the city to the urban agglomerations, and proposed measures for information coordination and collaborative construction of Information Infrastructure of urban agglomerations. Meanwhile, Shanghai, Nanjing, and Shenzhen pay more attention to micro-cultural development, propose Smart Communities, and implement pilot development of commercial districts and new town or villages to explore new models of community service. Nanjing is also concerned about the combination of tourism industry and its city cultural card with Smart” and strives to create a new bright spot of intelligence culture. Ningbo, as a port city, has focused its efforts on industrial development and comprehensive port services, with coordinated construction of seaport, land port, airport, and information port.

**Table 2:** Major initiatives for the development of typical Smart Cities in China

City	Correlative Policies and Regulations	Major Initiatives During the 13th Five-Year” Period
Beijing	“Smart Beijing Guideline for Action” “Beijing ‘13th Five-Year’ Information Development Plan”	Improving Information Infrastructure, optimizing IoT sensing facilities, and building big data and cloud computing infrastructure; constructing people-benefiting information service system and promoting convenient public services and smart services for people’s livelihood; promoting urban intellectualized management and realizing fine management, precise regulation, and accurate support; constructing fusion innovation environment and promoting innovation and entrepreneurship, integration and upgrading, and information industry development; implementing major strategic projects, including coordinated development of Beijing–Tianjin–Hebei informatization, and cyberspace security.
Shanghai	“Shanghai Action Plan for Promoting Smart City Development (2014–2016)” “Shanghai ‘13th Five-Year’ Plan for Promoting Smart City Development”	Building up a universalization of Smart City application development layout and creating a convenient Smart Life, constructing Smart Communities, villages, business districts, parks, and new cities; focusing on industrial innovation, developing a high-end Smart Economy, and accelerating the research and development and industrialization of a new generation of Information Technology; improving refined Smart Governance and building a collaborative wisdom government; improving the integrated wisdom city support system, accelerating Information Technology and related technology and service capacity enhancement, and focusing on building high-speed, mobile, and secure Information Infrastructure; enhancing network and information security guarantee capabilities and building an integrated support system of technology, industry, platform, and services
Guangzhou	“Guangzhou ‘12th Five-Year’ Science and Technology Development Plan (2011–2015)” “Guangzhou ‘12th Five-Year’ Information Development Plan (2011–2015)” “Guangzhou ‘13th Five-Year’ Information Development Plan (2016–2020)”	Coordinating the construction of new generation of Information Infrastructure and promoting the Pan-Pearl River Delta regional Information Infrastructure development; building a high-quality, high-end, and high-tech information industry system, promoting the development of big data industry and application innovation, and deepening the integration of traditional industries and the Internet; accelerating the development of new Smart City, establishing a comprehensive intelligent transportation system and energy system, improving the effectiveness of health care and education and cultural services, and improving the social security information service system
Nanjing	“Nanjing ‘12th Five-Year’ Smart City Development Plan” “‘13th Five-Year’ Smart Nanjing Development Plan”	Constructing the city’s intelligent portal and upgrading Nanjing intelligent command and operation center; improving information services for the people, enhancing social security services, and improving the effectiveness of public and government services; encouraging innovation of management mode, furthering the development of Smart Communities, and promoting the participation of social subjects in social governance; building the Nanjing Smart Tourism brand around the development concept of Internet + Tourism” and All-area Tourism”, which is supported by Smart Tourism data operation system, public tourism service platform, to promote the reconstruction of the tourism industry; creating new highlights of Smart Culture, highlighting Nanjing’s cultural business cards, and constructing the city’s comprehensive intelligent humanities service system; strengthening the development of Smart Environmental Protection, creating green low-carbon livable environment, and vigorously promoting energy recycling; playing the role of integration and interaction and promoting the rapid development of Smart Industry
Shenzhen	“Smart Shenzhen Planning Outline (2011–2020)” “Shenzhen informatization development ‘13th Five-Year’ plan”	Deepening the application of people’s livelihood, promoting quality and equal public services, promoting education informatization, medical informatization, and social security informatization, and building an integrated social security service system; accelerating the promotion of public culture, sports, and tourism informatization and building Shenzhen’s soft power; promoting the development of Smart Communities and exploring new modes of community management and services; strengthening urban wisdom management, building an intelligent municipal management system, implementing intelligent environmental protection and energy conservation, and building a Smart Transportation System. Building a Smart Transportation System and promoting Intelligent Urban Planning and Land Resource Management; implementing the Internet +” strategy, promoting the innovative development of the information economy and the integration of the Internet and industrial development, opening up new paths for industrial upgrading, and creating an innovation platform and an Internet innovation and entrepreneurship environment; innovating the application of big data, improving the government’s governance decision-making capabilities, promoting government governance change, and upgrading around government data opening and sharing applications; improving the basic support system, building a world-class information port, building an intelligent urban infrastructure perception system, strengthening security autonomy and control, and building an information security guarantee system
Ningbo	“Ningbo Smart City Development Master Plan” “Ningbo Smart City Development ‘13th Five-Year’ Plan”	Improving the integrated Information Infrastructure of sea, earth, and sky”, supporting the integrated development of seaports, dry ports, airports, and information ports, and upgrading the capacity of urban communication services; constructing a Smart City operation center, forming an urban big data ecosystem, and building a unified sharing platform for government data resources and an open platform for the city’s public data; accelerating the development of a comprehensive application system for Smart City, improving the level of urban management services, and promoting the development of smart social governance applications and smart life service applications; promoting the integration and innovative development of the wisdom industry, the transformation and upgrading of the urban economy, and the industrialization of smart applications and the industrialization of Information Technology; reinforcing the development of Smart City standards to achieve standardization of urban information space construction; enhancing information security of Smart City to ensure safe operation of the city

Source: City Government Portal Website

Typical cities focus on their correspondent goals of urban development, and actively promote the application of Information Technology in livelihood, transportation, healthcare, education, and e-government through the development in social, economic, spatial, and technological dimensions to achieve the smart development of the city. The development of typical cities provides experience for the development of other cities in China. However, small cities or less developed areas don’t have the chance to stand on the same starting line with developed areas in terms of infrastructure construction or informatization, because the level of development and the degree of informatization greatly vary between cities. Therefore, the focus of Smart City development in these cities or regions is also different from those of Beijing, Shanghai, Guangzhou, Nanjing, Shenzhen, Ningbo, and other cities that have better Information Infrastructure.

The Smart City should be built on the basis of technical support and widely adapt to social, political, economic, and cultural conditions. The focus and goals of different paths toward Smart City vary depending on the diverse aspects of city development, and the method of Smart City development is not exactly the same in various countries and cities. Therefore, the development of Smart Cities should be based on each city’s own specific conditions and development needs rather than copying the experience of other cities. Furthermore, a reasonable development direction should be chosen, a matching strategic positioning should be formulated, and the application of Information Technology in people’s livelihood, transportation, medical care, education, e-government, and other aspects should be actively promoted through the development of social, economic, spatial, and technical factors.

### Summary

China’s Smart Cities Development can be roughly divided into three stages. The first stage (2008–2012) was the start-up stage. This stage prioritized the introduction of the concept of Smart City, wireless communication, fiber optic broadband, GIS, GPS, RS, and other technologies. The enterprises were the first to spontaneously explore in this field and became the driver of this stage. The second stage (2012–2015) was the exploration stage. Governments in various regions responded and cooperated with equipment vendors and integrators, and cloud computing, SOA, 2G/3G/4G, and other Information Technologies were being used across the board, with the national ministries and commissions leading the development of pilot cities. The acceleration of urbanization not only brought economic support but also boosted the exploration of Smart Cities. This stage was driven by the government. The third stage (2016 to present) is the promotion stage. After the rapid development of the previous stage, the development of Smart Cities also began to slow down as the urbanization shifted to a new stage. The focus turns to people-oriented, integrated, and concentrated, with emphasis on effectiveness. Government-led development gives way to national coordination, government direction, market-led development, or PPP mode. Big data, artificial intelligence, blockchain, 5G, and other technologies have developed, allowing new business models to arise (China Academy of Information and Communication Research, 2019).

At present, the development of Smart Cities in China has been on the right track, although the technological development and applications are being refined and iterated. Nonetheless, the general focus is still on the development of technology, economy, and the development of Information Infrastructure, while the “value of data” is not sufficiently explored (Xu et al., 2012). Moreover, the attention to society, welfare, and equity is insufficient. Large gaps still persist. Currently, most of the Smart Cities is focusing on serving the government and boosting the economy, which helps the government achieve refined management; however, people and businesses are only exposed to a few applications. Although this issue was raised in the 2014 National New-type Urbanization Plan (2014–2020), and typical cities have responded in the 13th Five-Year Plan by focusing the development of Smart Cities on social governance, present efforts are still insufficient. The future of Smart City development should be extended to the civil and grassroots level, with the principle of “people-oriented”, to ensure that it can be used by the general public. Moreover, China’s regional development is uneven, and the foundation and level of digital economy and Smart City development vary from place to place, suggesting that the process of developing Smart Cities is not always the same and should be “a thousand cities and a thousand brains” (China Academy of Information and Communication Research, 2019). First-tier cities have already passed the threshold of Smart Information development and Smart Economy development and started to promote smart social governance. Meanwhile, other cities still need to focus on Information Infrastructure construction first to better promote other aspects of “Smart” in the future.

## Building the smart cities of the new era

Currently, China has entered a new stage of urbanization. Along with globalization, rapid industrialization has brought considerable dividends to China while leaving some social and environmental problems. For example, the change of land use from water area and grassland to the built-up environment results in the deterioration of the eco-environment and the declining of resources. Moreover, the continuous increase in urban scale is accompanied by the rising social heterogeneity, and the supply of social development and public services are unable to keep up with the accelerated economic and spatial urbanization. All these issues are significantly impeding sustainable development and urban quality improvement. While urbanization also offers new opportunities for growth, the innovation of a Smart City has inestimable power to resolve problems and may become vital to the concept of “Chinese Smart” in urban governance. As the greatest invention of humankind, cities carry the best hopes. Recognizing that an IT-based Smart City should propel society toward an improved urban civilization, China’s urbanization must immediately establish a “Smart City” of the new era.

### Constructing a rational, efficient and continuously innovative economic system

First and foremost, a Smart City is an economically healthy, science-based, and sustainable city. Central to its development is a rational, efficient, and innovative economic system. In the context of China, its economic development has entered a new normal, leading the city economy to encounter a new stage of strategic transformation. On top of that, the forthcoming technologically-enhanced “Smart” economic system should focus more on social equity, environmental ecology, and other life and livelihood issues while fostering new growth points in the city economy.

On the one hand, guiding the development of the Smart Industry necessitates an economic system tailored for a Smart City and aligned with China’s national conditions. The National New-type Urbanization Plan (2014-2020) proposes that the government should “respect the laws of the market more” while innovatively prioritizing the market over the government. This approach is beneficial to the Smart Industry for application experiments and operations. Meanwhile, the intervention of market forces also requires institutional protection. Stimulating the vitality and creativity of various markets and building an open and orderly modern market through appropriate government guidance will effectively promote a Smart Economic system.

On the other hand, open public platforms should be formed to stimulate innovation. Scientific and technological innovation motivates urban development, while market competition effectively drives the former. With the significant development of China’s market economy, open public platforms must be created to support industrial innovation, and collaborative innovation among industries, universities, and research institutes. Accordingly, this will (a) maximize the innovative backbone of large enterprises; (b) stimulate the innovative vitality of small and medium-sized enterprises; (c) promote the application of a more open public platform for the sharing of data, large equipment, and scientific research resources; and (d) integrate Smart Cities into the urban innovation system.

### Building a data-enabled, people-oriented social system

The social system comprises a series of behavioral activities among the people in the city. Through the support of “big data,” the building of a Smart Social System should always put the constant improvement of people’s livelihood in the first place and take serving the people and society as the main task. Given that China’s social development significantly lags behind its economic development, the value of fairness and justice should be further underscored. Therefore, using Smart Technology to enhance the quality of people’s lives should be one of the priorities of Smart City development to build a high-quality city with social sharing and provide long-term social support for economic development (Wang, 2012).

Establishing a Smart Government and offering public services are essential to the Smart Social System. Information sharing, resources integration, and business collaboration are required in Smart Government to achieve one-stop government services, as well as timely and accurate intelligent decision-making. Additionally, the expansion of supply capacity and innovation of supply model also required in public services supplement through the application of Information Technology, in order to highlight the core value and dynamic role of people, and to achieve an efficient, convenient, and intelligent supply of public products and services.

Furthermore, the principle of being “people-oriented” is re-emphasized. Informatization inevitably brings a digital divide and intensifies the differences between urban and rural areas. When building up the Smart Social System, resolving this “digital gap” should be prioritized in this case. On the one hand, the welfare of disadvantaged groups should be considered by ensuring the accessibility of Smart Services. As a result, they could fully enjoy the comfort, convenience, and services brought by a Smart City. On the other hand, the integration of the Internet and Communication Technology offers a platform for public participation. Then, open information and public participation could facilitate Smart Social Governance to realize positive social interaction and contribute to social progress.

### Develop smart urban agglomerations based on information and infrastructure sharing

Together with the urbanization process, the number and scale of cities in China are expanding, which has led to the formation of urban agglomerations. Nowadays, urban agglomerations have become the primary form of new-type urbanization. Its development encounters opportunities and challenges in various fields, such as those related to international communications, industrial transformation, regional integration, and the advancement of the human living environment. It is worth noting that with the application of Information and Communication Technologies, Smart City agglomerations are the advanced form of urban agglomerations, which provide new approaches to deepen the development of urban-rural integration and inter-city coordination.

First of all, fast and efficient information sharing is the basis of Smart Urban Agglomeration. Through the help of Smart Technology, a comprehensive and unified sensing network can be established to collect and integrate information in different fields, such as the employment, residence, healthcare, travel, education, and consumption of each city in the urban agglomeration. An intelligent management platform can also be formed with the help of Smart Technologies for information sharing, which effectively improves citizens’ quality of life and happiness in the urban agglomeration and making inter-city employment and life more convenient, fast, and intelligent.

Second, the sharing of regional infrastructure guarantees the development of Smart Urban Agglomerations. The national “new infrastructure strategy” has specified the direction for the shared construction of large regional infrastructure. More specifically, it involves the active development and enhancement of infrastructures for innovations in science and technology, education, medical care, pension, and other livelihoods and industries. Infrastructure services in terms of operational level and capacity can be extended to the largest regions using the Smart Technology, supporting the development of Smart Cities toward an integrated Smart Urban Agglomeration.

## Reflection and summary: towards a more intelligent urban society in China

Currently, the development of China’s Smart City is moving from conceptual development to multi-dimensional application, and Smart City or Digital City technologies, such as Internet, IoT, and cloud computing, are commonly promoted. Smart City includes the integration and application of various types of science and technology that deal with urban problems. However, technological progress is simply a necessity but not a sufficient condition for a Smart City, which should be a holistic vision of a better life for urban society and a comprehensive penetration from technology to “people-oriented” development. Therefore, considering the development of Smart Cities from a perspective beyond technological progress is a humanistic topic worth exploring under the current ever-changing technological wave, which can help in constructing a better Chinese urban society.

The core of urbanization is the urbanization of people, which is also the reflection of the high quality of urban society. The essence of developing Smart City is to use Information Technology to organically integrate new industrialization, informationization, urbanization, and agricultural modernization, to ensure that residents can have a comfortable, convenient, and healthy living environment, while also promoting the harmonious development of society. According to the report of the 19th National Congress, the goal of building a “Smart Society” is to expand and extend the width and depth of Smart Cities. It also reveals that the purpose of Smart City development is to use technology to make urban society develop in a better and healthier urban space and environment.

In conclusion, whether smart cities can make urban society better depends not only on the physical changes of the latest information technology on human life and spatial environment, but also on the social revolution of consciousness and philosophy, which will eventually realize a smarter Chinese urban society.

## Data Availability

Not Applicable.
